# Resuturing a Dislocated Scleral-Fixated Intraocular Lens in Brown–McLean Syndrome

**DOI:** 10.3390/jcm14165769

**Published:** 2025-08-14

**Authors:** Suguru Nakagawa, Atsushi Okubo, Kiyoshi Ishii

**Affiliations:** Department of Ophthalmology, Saitama Red Cross Hospital, Saitama 330-0081, Japan

**Keywords:** Brown–McLean syndrome, peripheral corneal edema, corneal endothelial cell loss, intraocular lens dislocation, intraocular lens resuturing

## Abstract

**Background/Objectives**: Brown–McLean syndrome (BMS) is a rare peripheral corneal edema that may arise years after cataract extraction or intraocular lens (IOLs) fixation. This article presents a case of IOL dislocation following scleral fixation in a patient with BMS, effectively managed by resuturing the existing IOL. Additionally, a literature review was conducted to summarize the clinical features, etiologies, and surgical outcomes of BMS. A PubMed search identified 30 reports encompassing 169 patients (244 eyes). Among these, corneal transplantation was performed in three eyes. Only four eyes underwent intraocular surgery after BMS onset, with no prior reports of IOL resuturing. **Methods**: A 73-year-old man with a history of left-eye trauma underwent vitrectomy and scleral fixation of a polymethyl methacrylate IOL 18 years prior. The patient presented with reduced vision in his left eye. Examination revealed BMS-related peripheral corneal edema and partial IOL dislocation. The dislocated haptic was resutured using an ab externo approach under a scleral flap. **Results**: Postoperative IOL fixation remained stable, with best-corrected visual acuity improving from 0.6 to 0.9. Edema persisted without central spread, and endothelial cell density decreased slightly (2496 to 2364 cells/mm^2^). One year postoperatively, no IOL tilt progression or suture-related complications were observed. **Conclusions**: Partial resuturing of a scleral-fixated IOL is effective for managing IOL dislocation in BMS when haptics remain stable. This approach minimizes incision size and potential endothelial trauma compared to explantation. However, aqueous dynamics correction may not reverse established BMS. Long-term endothelial monitoring is advised due to its chronic and progressive nature.

## 1. Introduction

Brown–McLean syndrome (BMS) is a rare condition characterized by peripheral corneal edema, typically occurring years after cataract surgery or intraocular lens (IOLs) implantation [1,2,3]. Although the exact pathogenesis remains unclear, BMS is recognized as a long-term complication of cataract surgery, particularly when associated with prolonged aphakia [1,2,4,5].

In cases of BMS, intraocular procedures, particularly IOL dislocation repairs, require caution, as surgical trauma to the corneal endothelium may further compromise its function and exacerbate peripheral corneal edema. To date, IOL resuturing in patients with BMS has not been specifically documented.

BMS is more common in aphakic eyes. In cases with prior iridectomy, a distinct “superior sparing” pattern and absence of edema in the superior cornea has been reported [6], suggesting aqueous humor dynamics in its pathogenesis.

This article presents a rare case of BMS developing 18 years after secondary scleral fixation of a rigid IOL, followed by delayed IOL dislocation. Unilateral IOL resuturing was performed, preserving central corneal clarity without BMS progression over 1 year. Furthermore, a review of the existing literature is included to contextualize this case and explore potential mechanisms and management strategies for this rare complication.

## 2. Materials and Methods

### 2.1. Patient and Examination

**Figure 1:**
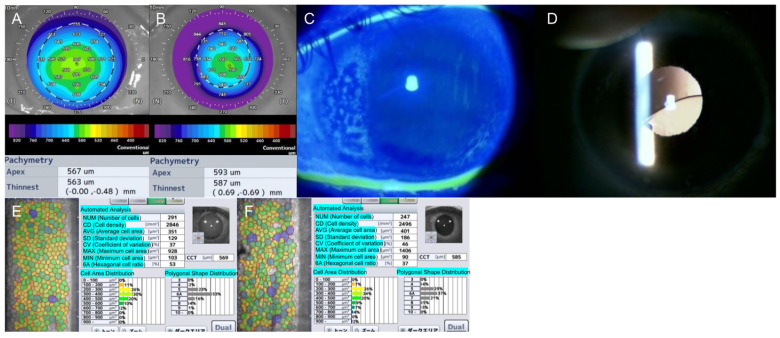
Preoperative findings. (**A**) Corneal pachymetry obtained via anterior segment optical coherence tomography (OCT) of the right (fellow) eye. (**B**) Corneal pachymetry of the left (affected) eye. (**C**) Slit-lamp photograph showing peripheral corneal edema, a characteristic finding of Brown–McLean syndrome, captured under fluorescein staining. The peripheral cornea exhibits edema and shows fluorescein uptake due to epithelial swelling. (**D**) Slit-lamp photograph showing intraocular lens (IOLs) decentration. Although the temporal (ear-side) suture remains intact, the nasal haptic appears to have become unsecured. Specular microscopy images showing ECD of the right (fellow) eye (**E**), while the left (affected) eye is depicted in image (**F**).

In June 2021, a 73-year-old man presented with gradual vision loss in his left eye. The ocular history included trauma followed by vitrectomy and scleral fixation of a polymethyl methacrylate intraocular lens (IOL, CZ70BD, Alcon Laboratories, Inc. Fort Worth, TX, USA) in 2003. Preoperative assessment revealed a best-corrected visual acuity of 0.6 in the left eye (Figure 1).

Anterior segment optical coherence tomography (AS-OCT, CASIA2, Tomey Corporation, Nagoya, Aichi, Japan) of the right (healthy) eye revealed normal corneal thickness (Figure 1A), while the left (affected) eye exhibited a central corneal thickness of 593 μm with marked peripheral edema (Figure 1B), confirmed by fluorescein staining (Figure 1C). The edema was circumferential, consistent with BMS (Figure 1B,C). Slit-lamp examination revealed partial IOL dislocation (Figure 1D), with the inferior haptic displaced into the vitreous cavity, while the superior haptic suture remained intact.

Specular microscopy indicated that the central endothelial cell density (ECD) was relatively preserved in both eyes (Figure 1E,F). Notably, the left eye maintained a density of 2496 cells/mm^2^ centrally (Figure 1F), despite peripheral edema consistent with BMS.

### 2.2. Surgical Procedure

**Figure 2:**
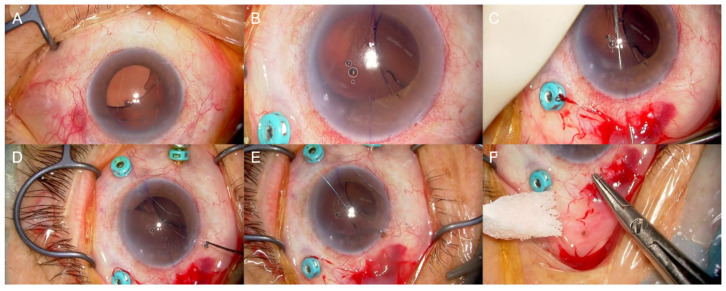
Intraoperative findings of intraocular lens (IOLs) resuturing. (**A**) Initial intraoperative image demonstrating circumferential peripheral corneal edema consistent with Brown–McLean syndrome. The upper part of the photograph corresponds to the inferior aspect of the eye. (**B**) The dislocated nasal-inferior haptic (at the 8:30 clock position) is retrieved from the eye through a 7 o’clock side port. The top of the image is the temporal side (applies to **B**–**F**). (**C**) A straight 9-0 polypropylene needle is inserted approximately 2 mm posterior to the limbus and guided across the anterior chamber to exit through the opposite side port using a “needle-retrieval” technique. (**D**) The 9-0 polypropylene needle is pulled out through the 7 o’clock side port. (**E**) The 9-0 polypropylene suture is externally secured to the eyelet of the IOL haptic. (**F**) The suture is anchored beneath a partial-thickness scleral flap created at the 8:30 position, and both the scleral flap and conjunctiva were closed.

Circumferential peripheral corneal edema consistent with BMS was observed preoperatively (Figure 2A) and documented in the video (Video S1). Under retrobulbar anesthesia, a 25-gauge pars plana trocar was placed 3.5 mm posterior to the limbus to access the dislocated haptic and facilitate posterior segment evaluation. A partial-thickness scleral flap was created at the 8:30 position.

A 25-gauge vitreous forceps was introduced through a side port at the 7 o’clock position to grasp the inferiorly dislocated haptic, which was carefully extracted through the same port (Figure 2B, Video S1). Subsequently, the straight needle of a 9-0 polypropylene suture (Mani Co., Ltd., Utsunomiya, Tochigi, Japan. catalog #1494P), connected to a curved needle, was inserted through the scleral flap 2 mm posterior to the limbus at the 8:30 position. A 27-gauge guiding needle was introduced via the contralateral side port to retrieve and extract the straight needle (Figure 2C, Video S1).

The 9-0 polypropylene suture, traversing the anterior chamber, was externally retrieved through the 7 o’clock side port using a hook (Figure 2D, Video S1). After sectioning the suture, the end connected to the scleral flap (with the curved needle) was threaded through the haptic eyelet and securely fastened (Figure 2E, Video S1). The opposite end, initially introduced with the straight needle and retrieved through the contralateral port, was excised. The haptic was repositioned intraocularly, and the suture tension was adjusted to achieve optimal IOL centration in the posterior chamber. Finally, the suture was anchored beneath the scleral flap using the curved needle, followed by closure of both the scleral flap and conjunctiva (Figure 2F, Video S1).

Since the superior haptic remained securely fixed, no additional manipulation was required. Finally, the viscoelastic material was then thoroughly irrigated from the anterior chamber using balanced salt solution, concluding the procedure. Postoperative topical steroids, such as prednisolone acetate 1%, were prescribed alongside antibiotic drops. Follow-up evaluations included slit-lamp biomicroscopy, AS-OCT, and specular microscopy to assess corneal status and IOL stability.

## 3. Results

**Figure 3:**
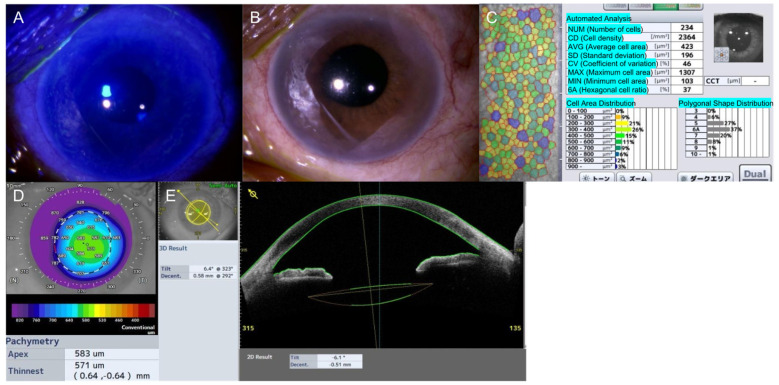
Postoperative follow−up at 3 months and 1 year. (**A**,**B**) Slit−lamp photographs at 3 months postoperatively showing peripheral corneal edema consistent with Brown−McLean syndrome. Edema and fluorescein staining from epithelial swelling remain stable compared with preoperative findings. (**C**) Specular microscopy image showing the corneal endothelial cell status at 3 months postoperatively. (**D**) Anterior segment optical coherence tomography (OCT) corneal pachymetry of the left (affected) eye at 1 year postoperatively. (**E**) Anterior segment OCT showing the intraocular lens (IOLs) fixation status at 1 year after surgery.

At one week postoperatively, the IOL remained well-centered, with no signs of significant inflammation. By 1 month, best-corrected visual acuity (BCVA) improved to 0.7, while peripheral corneal edema remained stable. At 3 months (Figure 3A–C), BCVA reached 0.9. Slit-lamp photographs revealed persistent peripheral corneal edema, consistent with BMS, without central corneal involvement (Figure 3A,B). Central ECD remained stable at 2364 cells/mm^2^, representing a 5.3% decrease from baseline (2496 cells/mm^2^; Figure 3C). By 1 year (Figure 3D,E), peripheral corneal edema persisted without central involvement (Figure 3D). The IOL remained well-positioned, exhibiting minimal tilt (6.1°), slight decentration (0.51 mm), and no suture-related complications (Figure 3E).

## 4. Discussion

This article presents a case of BMS with late-onset IOL dislocation occurring 18 years after scleral fixation. The dislocated IOL was successfully resutured, resulting in a favorable outcome. At the 1-year follow-up, central ECD and corneal clarity were preserved, with no progression of peripheral corneal edema.

BMS is a rare condition characterized by peripheral corneal edema that develops years to decades after intraocular surgery, including cataract extraction or IOL implantation [1,2,4,5,6]. First described by Brown and McLean in 1969, BMS has been reported in 169 cases (244 eyes) across 30 English-language articles retrieved via a PubMed search using “Brown–McLean” as a keyword on 3 May 2025 (Table 1). Intracapsular cataract extraction is the primary antecedent procedure in BMS. Other associated surgical backgrounds include extracapsular cataract extraction, phacoemulsification, anterior chamber IOL implantation, scleral-fixated IOL insertion, congenital cataract surgery, aphakia following trauma, and various glaucoma procedures (Table 1). Reports of intraocular surgery in eyes with BMS remain limited. Among the 244 eyes documented, only four underwent additional intraocular procedures following BMS onset (Table 2). In all cases, central corneal clarity was preserved without disease progression. Notably, reports describing IOL refixation in BMS are extremely rare. This case presents a valuable clinical observation, demonstrating that unilateral IOL resuturing in a patient with BMS can enhance visual function while preserving central corneal transparency.

In patients with BMS requiring intraocular surgery, such as IOL dislocation repair, meticulous surgical technique is essential to prevent intraoperative trauma to the corneal endothelium. Preserving endothelial integrity is critical for minimizing cell loss, limiting peripheral corneal edema progression, and maintaining central corneal clarity. In this case, the dislocated IOL was a rigid, single-piece polymethyl methacrylate (PMMA) lens, necessitating a large (approximately 7 mm) corneoscleral incision for explantation. Furthermore, the superior haptic was firmly fixated, and forced removal posed a risk of zonular or ciliary body injury. Additionally, severing the rigid PMMA haptic carried the potential for intraocular and endothelial damage. Considering these risks, IOL exchange was deferred in favor of resuturing a single haptic of the existing lens, thereby minimizing surgical invasiveness and further endothelial cell loss. Over a 12-month follow-up, both IOL positioning and visual acuity remained stable. The central ECD slightly decreased from 2496 to 2364 cells/mm^2^ (−5.3%) in 3 months, with no evidence of peripheral corneal edema progression.

The observed 5.3% decrease in central ECD at 3 months (from 2496 to 2364 cells/mm^2^) falls within reported ranges after scleral fixation—~6.0% [31] at 3 months with the Yamane technique and a pooled mean of ~8.95% [32] across SFIOL procedures at ~17 months—suggesting acceptable endothelial safety in this case. Although ECD was not reassessed beyond 3 months, anterior segment OCT showed stable central corneal thickness through 12 months without central edema progression; thus, in a BMS eye, resuturing did not appear to accelerate central endothelial loss beyond typical postoperative changes. Given persistent peripheral edema and physiologic age-related decline (~0.3–0.6%/year) [33], long-term endothelial surveillance remains warranted.

### 4.1. Technique Selection for a Rigid PMMA IOL

In the present case, the dislocated lens was a rigid single-piece PMMA IOL. Flange-based sutureless intrascleral haptic fixation (e.g., the Yamane technique) is not suitable for this lens design, and explantation would have required a 6–7 mm wound with increased anterior segment manipulation, both unfavorable in a BMS eye with limited endothelial reserve. Because the fellow haptic remained well secured, we prioritized endothelial safety and performed ab externo unilateral haptic resuturing, minimizing intraocular time and avoiding wound enlargement. This approach achieved stable centration and visual recovery without central edema progression.

### 4.2. Alternative Options Applicable to Rigid PMMA

Two additional strategies can “resuture” a rigid PMMA IOL while preserving the existing lens: Canabrava’s four-flanged polypropylene fixation [34]—typically using 5-0 polypropylene (with 6-0 variants described) to create four flanges for knotless scleral fixation, with careful matching of flange size to scleral tunnel caliber—and Gore-Tex (ePTFE) sutured fixation using a Hoffmann pocket [35], which offers robust buried-knot support but requires specific suture availability and entails suture-related long-term considerations.

### 4.3. Comparative Rationale vs. Exchange in Foldable Lenses

If the displaced lens had been foldable, small-incision IOL exchange followed by sutureless intrascleral fixation (e.g., Yamane [36,37,38,39,40]) or four-point fixation would have been reasonable alternatives that may reduce late suture-related events (loosening/breakage) and can shorten operative time in selected scenarios. These benefits must be balanced against potential trade-offs—such as IOL tilt, decentration, or iris capture—determined by haptic geometry and the fixation method.

### 4.4. Practical Framework

We individualize the choice according to (i) IOL material/design and optical integrity (rigid PMMA vs. foldable; clarity/damage), (ii) endothelial safety (especially in BMS), (iii) the need to minimize wound size and anterior segment manipulation, and (iv) equipment/implant availability and surgeon’s expertise. Applied to this patient, these factors favored unilateral ab externo resuturing over exchange.

Late IOL dislocation can be facilitated by multiple factors, including zonular weakness (age-related or pseudoexfoliation-related), ocular trauma, inflammation, prior pars plana vitrectomy (PPV), and lack of capsular support. In the present case, there was no clinical evidence of pseudoexfoliation in either eye, and the fellow eye showed no signs suggestive of zonulopathy. By contrast, the patient had a history of ocular trauma, developed rhegmatogenous retinal detachment, underwent PPV with phacoemulsification, and subsequently required repeat PPV with an encircling procedure for redetachment. After approximately 1 year of aphakia, a scleral-sutured IOL was placed, i.e., there was no in-the-bag or sulcus support from the outset.

Taken together, the currently observed late partial IOL dislocation after scleral fixation most plausibly reflects a multifactorial mechanism—involving late suture loosening/fatigue in a scleral-sutured IOL, compounded by multiple posterior segment surgeries and absence of capsular support—rather than pseudoexfoliation-related zonulopathy. In this case, IOL dislocation occurred 18 years after scleral fixation, and the eye exhibited the clinical phenotype of BMS.

The etiology of BMS remains uncertain, but a combination of genetic predisposition and risk factors—including prior intraocular surgery, glaucoma, and myotonic dystrophy—has been proposed (Table 1) [6,13,27]. BMS has been reported in patients with postsurgical complications and multiple intraocular procedures, with a higher frequency in aphakic eyes (Table 1) [6,27]. Reported onset typically occurs 6–16 years after surgery, with cases up to 33 years [2]; the 18-year interval in our case is therefore within the published range.

The corneal phenotype—persistent peripheral edema with central sparing—is consistent with BMS and aligns with patterns described in the literature. Overall, this case represents a typical constellation of BMS-associated risk factors rather than a unique presentation.

One of the key considerations in this case is whether IOL dislocation contributed to the onset of BMS. Although several theories have been proposed, no single pathophysiological mechanism for BMS has been confirmed; the following remain hypotheses. Several mechanisms have been proposed for its pathogenesis, including (a) mechanical trauma from iridodonesis, potentially induced or exacerbated by altered aqueous humor dynamics [2,5,7,8,9,13,15,18]; (b) chronic micro-iridodonesis or mechanical irritation of the iris by the IOL, potentially leading to low-grade iritis and subsequent peripheral corneal endothelial dysfunction, thereby contributing to recurrent corneal edema [6,14]; and (c) impaired peripheral migration of corneal endothelial cells due to physical barriers such as Schwalbe’s line-like structures or degenerated cells located in the inner peripheral cornea, resulting in localized endothelial cell deficiency and peripheral corneal edema [10].

The characteristic “superior sparing” pattern—edema sparing the superior cornea near the iridectomy—further supports the role of iridodonesis and flow disturbances in the pathogenesis of BMS [6]. In this case, the dislocated IOL may have altered local aqueous humor dynamics, exacerbating iridodonesis and contributing to BMS progression. Furthermore, micro-iridodonesis or mechanical irritation of the iris by the dislocated IOL may have induced low-grade iritis, potentially triggering or worsening BMS. If this hypothesis is valid, restoring physiological aqueous flow through IOL resuturing might help mitigate disease progression or alleviate symptoms. However, no significant improvement in peripheral corneal edema was observed during the 1-year postoperative follow-up. Tomioka et al. [28] proposed that Schwalbe’s line-like structures in the peripheral cornea or degenerated cells acting as physical barriers may prevent corneal endothelial cell migration from the central area. If this hypothesis holds true, enhancing aqueous flow alone may be insufficient to promote BMS recovery, possibly explaining the lack of improvement following IOL resuturing in this case. Moreover, a 1-year observation period may be inadequate to fully assess the impact of IOL resuturing on BMS progression and central corneal endothelial transparency. Longer-term follow-up and accumulation of comparable cases will be essential to advancing clinical understanding.

Among the 169 reported cases (244 eyes) of BMS in the literature (Table 1), corneal transplantation was required in only one eye described by Swan et al. [6] and two eyes reported by Reed et al. [4]. In most instances, the central corneal endothelium remains intact, and keratoplasty is rarely indicated. For example, Tomioka et al. [28] documented a minimal decrease in central ECD over 12 years, from 2499 cells/mm^2^ to 2456 cells/mm^2^, corresponding to an annual cell loss rate of only 0.09%.

Nevertheless, rare progressive cases necessitating corneal transplantation have been reported, highlighting the importance of regular follow-up, including periodic endothelial assessments in patients with BMS.

In recent years, the incidence of IOL dislocation has risen, partly due to advances in cataract surgery and an aging population [36,37,38,39]. Furthermore, postoperative complications such as iris fluttering and iris capture following IOL dislocation or scleral fixation are not uncommon [37,38,39,41] and may contribute to BMS development. Given that iridodonesis in aphakic eyes lacking zonular support is a proposed mechanism underlying BMS, its prevalence may increase in the future. Therefore, enhanced recognition and understanding of BMS will be essential for optimizing long-term ophthalmic care.

### 4.5. Limitations

The 12-month follow-up in this report is insufficient for a chronic condition such as BMS. Longer-term surveillance (≥3 years) is warranted to evaluate late complications, including suture fatigue, IOL tilt or re-dislocation, progressive endothelial cell loss, and peripheral edema progression. After 12 months, the patient is transitioned to follow-up at a local clinic; to date, no re-presentation or complication reports have reached our center. Nevertheless, firm conclusions cannot be drawn without extended follow-up.

## 5. Conclusions

In patients with BMS and partial IOL dislocation, unilateral resuturing of the existing IOL offers a safe, minimally invasive approach for restoring visual function while preserving central corneal clarity. When a rigid PMMA IOL is already fixated, this approach mitigates the risks associated with explantation and large corneoscleral incisions. Although repositioning did not improve peripheral corneal edema in this case, the procedure did not exacerbate BMS, suggesting that aqueous humor dynamics alone may not determine disease progression. This case highlights the feasibility and safety of IOL resuturing, even in eyes affected by BMS. While resuturing can restore lens position and visual function, it may not halt the progression of peripheral edema in BMS; therefore, lifelong endothelial surveillance is warranted.

## Figures and Tables

**Table 1:** Summary of 30 publications on Brown–McLean syndrome (1969–2025): A total of 169 cases and 244 eyes.

Author(s)	Year	Number of Patients (Eyes)	Age (Years)	Sex (M/F)	Time to BMS Onset (Years)	Previous Surgeries	Underlying Conditions
Brown SI & McLean JM [1]	1969	8 (14 eyes)		2/6	>6	ICCE	
Brown SI [7]	1970	5 (10 eyes)		0/5	>6	ICCE (8 eyes)/ECCE (1 eye); spontaneous superior lens subluxation (1 eye)	Marfan syndrome
Charlin R [8]	1985	16 (26 eyes)		8/8	14.5	ICCE (21 eyes)/ECCE (3 eyes); spontaneous lens absorption (2 eyes)	High myopia (61%); 1 trauma; 1 congenital rubella
Lim JI et al. [9]	1991	1 (1 eye)	52	1/0	34	ICCE	Trauma (traumatic cataract)
Tuft SJ et al. [2]	1992	15 (21 eyes)	63 (range: 32–81)	7/8	13.5 (range: 1–33)	ICCE (17 eyes)/ECCE (2 eyes); lens aspiration (2 eyes)	RD (4 eyes); high myopia (3 eyes); prior PPV (2 eyes); 1 uveitis; 1 glaucoma; 1 iris prolapse
Reed JW et al. [4]	1992	22 (36 eyes)		10/12		ICCE (34 eyes)/ECCE (2 eyes)	
Gothard TW et al. [5]	1993	32 (43 eyes)	69.6 (range: 26–88)	18/14	15.6 (range: 1.5–30)	ICCE ± superior PI (31 eyes); ECCE (7 eyes); PEA (2 eyes); LI (2 eyes); PPL (1 eye)	2 eyes: intermittent angle-closure glaucoma
Hara T et al. [10]	1993	1 (1 eye)	65	0/1	3.5	ECCE with iris-fixated IOL (Binkhorst)	None
Sugar A [11]	1997	1 (2 eyes)	76	0/1	RE:15 LE:16	ICCE/ECCE; PKP; anterior vitrectomy; iris-supported IOL; IOL explantation	Bilateral uveitis ± CME; chronic iritis; vitreous hemorrhage
Sun B et al. [12]	1998	5 (8 eyes)			10.5	Cataract surgery	
Rutzen AR et al. [13]	2001	1 (2 eyes)	50	1/0		None	Myotonic dystrophy
Vote BJ et al. [14]	2003	3 (6 eyes)	Case 1: 45, Case 2: 46, Case 3: 68	1/2		None (Case 1); bilateral PPV + lensectomy + AC-IOL (Case 2 and RE of Case 3); lens subluxation (untreated, LE of Case 3)	Familial predisposition (visual impairment/tendon rupture)
Martins EN et al. [15]	2004	2 (2 Eyes)	Case1 62, Case2: 51	0/2	Case 1: 30 Case 2: 17	None (traumatic lens dislocation)/presumed ICCE	Trauma/Glaucoma; posterior staphyloma
Almousa R et al. [16]	2007	2 (2 Eyes)	Case 1: 79, Case 2: 12	0/2	Case 1: 17 Case 2: 11	ICCE + superior PI; congenital cataract aspiration	Uveitis (fellow eye)/None
Diaz-Llopis M et al. [17]	2007	10			1.5–4.5	Refractive phakic AC IOLs	
Vogel MS et al. [18]	2011	2 (4 Eyes)	Case 1: 78, Case 2: 82	2/0	Case 1: 37–38 Case 2: 30	Bilateral ICCE + PI ± secondary IOLs	Glaucoma; hypertension; prostatic hypertrophy/ diabetes; dyslipidemia
Lim LT et al. [3]	2012	1 (2 eyes)	78	1/0	17	Bilateral ICCE + superior PI	None
Jamil AZ et al. [19]	2012	1 (1 eye)	30	0/1		None	Bilateral keratoconus; non-systemic
Kam KW et al. [20]	2013	1 (1 eye)	80	1/0	13	ICCE + superior PI	High myopia; non-systemic
Mai HT et al. [21]	2013	1 (1 eye)	87	1/0	15	ERM surgeries; cataract surgery (unspecified)	None
Tourkmani AK et al. [22]	2015	1 (2 eyes)	12	1/0	11	Bilateral PEA + PCIOL in infancy	Congenital cataract; amblyopia/nystagmus; non-systemic
Suwan Y et al. [6]	2016	28 (35 eyes)	45 (range: 18–80)	12/16		Mainly ICCE (11 eyes); couching (5 eyes); lens aspiration/PEA/lens dislocation w/o surgery (4 eyes)	Marfan syndrome; familial iris hypoplasia; high myopia (40%)
Mohebbi M et al. [23]	2016	1 (2 eyes)	31	0/1	25	Bilateral lensectomy (aphakia); iris-claw IOL (RE)	Hallermann-Streiff syndrome
Mallikarjun MH et al. [24]	2019	1 (1 eye)	73	1/0	8	PEA + PCIOL (RE); contralateral untreated	None
Chatterjee S et al. [25]	2020	1 (1 eye)	52	1/0	11	Bilateral cataract surgery without IOLs	Pathologic high myopia; ERM/macular pucker
Alenezi SH et al. [26]	2020	1 (1 eye)	29	1/0	>20	Bilateral PPV + lensectomy; SB + cryo (LE)	Homocystinuria with lens dislocation; glaucoma
Alburayk K et al. [27]	2023	1 (2 eyes)	35	1/0		Childhood bilateral trabeculectomy	Congenital glaucoma; non-systemic
Tomioka Y et al. [28]	2024	1 (1 eye)	72	1/0	35–40	Multiple PPVs for RD; aphakia with iris damage	RD; postoperative iris damage; aphakia
Guedes J et al. [29]	2024	3 (5 eyes)	Case 1: 26, Case 2: 55, Case 3: 74	3/0	Case1: 21 Case2: 50 Case 3; appx. 20–30	Pediatric cataract surgeries (5 eyes); RD surgery (2 eyes)	Microphthalmos; macular scar; family history of Fuchs dystrophy; high myopia; RD history
Wan Q et al. [30]	2025	1 (1 eye)	18	1/0		None	Myopia; non-systemic; no contact lens use

Abbreviations: ICCE = intracapsular cataract extraction; ECCE = extracapsular cataract extraction; PEA = phacoemulsification aspiration; PI = peripheral iridectomy; PPL = pars plana lensectomy; LI = laser iridotomy; AC-IOL = anterior chamber intraocular lens; PC-IOL = posterior chamber intraocular lens; SFIOL = scleral-fixated intraocular lens; RD = retinal detachment; RE = right eye; LE = left eye; FHx = family history; PKP = penetrating keratoplasty.

**Table 2:** Reported cases of intraocular surgery in eyes with pre-existing Brown–McLean syndrome and their postoperative outcomes (among 244 eyes reported in the literature).

Author(s)	Year	Previous Surgeries	Underlying Conditions	Age (Years)	Sex	Surgical Intervention	Postoperative BMS Course
Rutzen AR et al. [13]	2001	None	Cataract, Myotonic dystrophy, central guttae	50	M	PEA + IOL	Peripheral edema unchanged, central clarity maintained
Vote BJ et al. [14]	2003	None	Aphakia (vitreous dislocation of crystalline lenses).	45	F	SFIOL+ PPV + lensectomy	Peripheral edema unchanged, central clarity maintained
Kam KW et al. [20]	2013	ICCE + superior iridectomy (1981)	Aphakia, High myopia	80	M	Aphakia after ICCE in 1981, SFIOL in 2001, replaced by SFIOL in 2007	Peripheral edema unchanged, central clarity maintained
Mohebbi M et al. [23]	2016	Bilateral lensectomy at age 6	Aphakia, Hallermann-Streiff syndrome	31	F	Artisan IOL implanted	Transient worsening of edema post-op, improved in 2 days, stable at 3 months
Nakagawa S et al. (This case)	2025	Ocular trauma; PPV; secondary SFIOL	IOL dislocation	73	M	SFIOL resuturing	Peripheral edema unchanged, central clarity maintained at 1 year

Abbreviations: ICCE = intracapsular cataract extraction; PEA = phacoemulsification aspiration; SFIOL = scleral-fixated intraocular lens; Artisan IOL = iris-claw intraocular lens; BMS = Brown–McLean syndrome.

## Data Availability

The data generated in this study are available from the corresponding author upon reasonable request.

## Supplementary Materials

The following supporting information can be downloaded at https://www.mdpi.com/article/10.3390/jcm14165769/s1, Video S1: Surgical resuturing of a dislocated scleral-fixated IOL in a case of Brown–McLean syndrome.

## Abbreviations

The following abbreviations are used in this manuscript:

AS-OCT	Anterior segment optical coherence tomography
BCVA	Best-corrected visual acuity
BMS	Brown–McLean syndrome
IOL	Intraocular lens
PMMA	Polymethyl methacrylate
